# Health economic evaluation of hip fracture care pathways in The Gambia

**DOI:** 10.1136/bmjgh-2025-022219

**Published:** 2026-09-30

**Authors:** Nyashadzaishe Mafirakureva, Kebba S Marenah, Anya Burton, Tafadzwa Madanhire, Hannah Wilson, Momodou K Jallow, Awa Touray, Lucy Gates, Bintou Trawally, Simon Matthew Graham, James Masters, Matthew L Costa, Kate A Ward, Celia L Gregson, Sian M Noble

**Affiliations:** 1 Health Economics & Decision Science, The University of Sheffield, Sheffield Centre for Health and Related Research, Sheffield, UK; 2 Musculoskeletal Research Unit, Translational Health Sciences, University of Bristol, Bristol, UK; 3 Department of Orthopaedics & Trauma, Edward Francis Small Teaching Hospital, Banjul, Gambia; 4 Musculoskeletal Research Unit, Translational Health Sciences, University of Bristol Medical School, Bristol, UK; 5 MRC Unit The Gambia at LSHTM, Banjul, Gambia; 6 Medical Research Council Unit The Gambia at the London School of Hygiene and Tropical Medicine, Banjul, Gambia; 7 School of Healthcare Innovation & Enterprise, Faculty of Medicine, University of Southampton, Southampton, UK; 8 Nuffield Department of Orthopaedics, Rheumatology and Musculoskeletal Sciences, University of Oxford, Oxford, UK; 9 Oxford Trauma, Nuffield Department of Orthopaedics, Rheumatology, and Musculoskeletal Sciences, Oxford University, Oxford, UK; 10 The Health Research Unit Zimbabwe, Biomedical Research and Training Institute, Harare, Zimbabwe; 11 Population Health Sciences, Bristol Medical School, Bristol, England, UK

**Keywords:** Africa, Aging, Global Health, Orthopedic surgery, Hospital-based study

## Abstract

Hip fractures cause substantial morbidity, mortality and healthcare costs among older adults. While advances in surgical and postoperative care have improved outcomes in high-income countries, these interventions are often inaccessible in low-income and middle-income countries due to cost constraints. This study assessed the costs and outcomes of surgical versus non-surgical management of hip fractures to inform resource allocation in a resource-limited setting. Data from a prospective cohort study of patients with acute hip fractures over a 12-month postfracture follow-up period were used. Outcomes included quality-adjusted life years (QALYs), healthcare resource use and costs in 2024 US$. Differences in costs and QALYs were estimated using multivariable regression analysis, while a range of sensitivity analyses assessed the impact of different assumptions. The analysis included 137 patients with a mean age of 70 years (SD: 13.4); 60% (n=82) were female and 81.8% (n=112) had fragility fractures. Fewer than half (47%, n=64) had surgery. Surgical management incurred US$988 higher costs (95% CI US$843 to US$1132) and generated 0.122 additional QALYs (95% CI −0.034 to 0.277) compared with non-surgical care, yielding an incremental cost-effectiveness ratio (ICER) of US$8117 per QALY gained. The probability of surgery being cost-effective exceeded 50% at willingness-to-pay thresholds of US$8200 per QALY gained or higher. The results were sensitive to surgical timing: operating within 3 days of admission more than doubled health gains to 0.247 QALYs (95% CI 0.056 to 0.439) and reduced the ICER to $4974 per QALY gained compared with no surgery. In this resource-limited setting, surgical management of hip fractures is more expensive but delivers clinically meaningful benefit for patients; a gain of 0.12 QALYs per patient translating to an extra 44 days in good health. Health systems in resource-limited settings should prioritise expanding surgical access while optimising early intervention and postoperative care to maximise benefits.

**WHAT IS ALREADY KNOWN ON THIS TOPIC**

- Hip fractures in older adults cause high morbidity, mortality and costs, yet surgical care remains largely inaccessible in low-resource settings.

**WHAT THIS STUDY ADDS**

- This is the first economic evaluation of hip fracture care in The Gambia and West Africa.
- Surgery increased costs but provided meaningful health gains (0.12 quality-adjusted life years (QALYs) ≈ 44 extra days in good health) compared with no surgery.

**HOW THIS STUDY MIGHT AFFECT RESEARCH, PRACTICE OR POLICY**

- Findings support the case for investing in surgical capacity to provide timely hip fracture care in The Gambia and similar low- and middle-income countries (LMIC) settings.
- Results can guide policy decisions on the expansion of both the National Health Insurance Scheme and National Trauma and Injury Plan in The Gambia.

## Introduction

 The demographic transition in Africa is leading to an increasing proportion of older individuals, with projections indicating that the continent’s population aged 60 and above will triple by 2050.^1 2^ This shift is expected to drive a surge in age-related health conditions, including osteoporosis and fragility fractures.^3 4^ Hip fractures, in particular, are associated with high morbidity, mortality and healthcare costs. Recent epidemiological studies from South Africa and Zimbabwe suggest that hip fracture incidence will double over the next 2.5 decades if preventive and therapeutic measures are not implemented.^5 6^ Urgent measures are required to attenuate this increase and prepare healthcare systems to manage this emerging burden.

The Gambia is a low-income country, with a gross domestic product (GDP) per capita of US$908 in 2024 and healthcare spending of US$28 per capita in 2022.^7^ The country’s healthcare system is characterised by limited infrastructure, workforce shortages and financial constraints. Orthopaedic care is particularly under-resourced, with less than one orthopaedic surgeon available for 100 000 adults and only four facilities (one public) nationally providing surgical management for fractures.^8^ Importantly, traditional bone setters (TBS) play a significant role in fracture care, often serving as the first point of contact for patients due to cultural beliefs, accessibility and affordability.^8 9^ However, non-surgical management, including care by TBS, has been associated with increased risk of complications, poor health outcomes and mortality.^9–11^

The anticipated rise in hip fracture rates, combined with existing constraints in The Gambia’s healthcare system, creates an urgent need to evaluate the economic implications of different care pathways. Surgical intervention is widely regarded as the standard of care for hip fractures, as it alleviates pain and restores mobility. However, in low-resource settings, the health outcomes, costs and overall cost-effectiveness of surgical management remain uncertain. Evidence from our research in African contexts shows that while surgery leads to substantial improvements in health-related quality of life relative to non-surgical management,^12^ it does so at a substantially higher cost.^12 13^

This study aims to compare the costs and outcomes of surgical versus non-surgical management of hip fractures in The Gambia. The findings will provide critical evidence to inform the design of appropriate care pathways and optimise context-appropriate resource allocation within the country’s healthcare system. The recent introduction of The Gambia’s National Health Insurance Scheme (NHIS),^14^ which currently covers only a few public hospitals and a limited set of services, provides a timely opportunity. Findings from this study can guide the scheme in prioritising service expansion and ensuring that coverage decisions are grounded in evidence of both clinical benefit and economic value.

## Methods

The analysis is based on a prospective cohort study of patients with acute hip fractures over a 12-month postfracture follow-up period. The analysis was conducted from a healthcare sector perspective, which considers and counts all ‘formal healthcare sector (medical) costs borne by third-party payers or out-of-pocket costs paid by patients’.^15^ During the study period (2021–2023), formal health insurance coverage in The Gambia was minimal, as the NHIS was in its early phased rollout. While primary public health consultations involve nominal user fees, specialised surgical hardware and supplies require direct out-of-pocket payments. TBS are frequently the primary fracture care providers in The Gambia’s *pluralistic* healthcare system,^8^ particularly in rural areas with limited access to formal medical facilities. Although operating outside the regulated healthcare sector, this analysis intentionally incorporates TBS within the formal healthcare sector due to their widespread use in this setting. The analysis considered costs and health outcomes during the first year after fracture only, hence no discounting was applied.

### Population and setting

A prospective cohort study was conducted as part of the Fractures-E3 (Fractures in sub-Saharan Africa: epidemiology, economic impact and ethnography) research programme, with the protocol previously reported.^16^ The study population included men and women aged ≥40 years sustaining/presenting with a new hip fracture and residing in the urban/peri-urban areas of Banjul, Kanifing and Brikama, and the rural West Kiang area. Patients presenting with a hip fracture to a study hospital (public or private) and/or a TBS were invited to participate. Within the study areas, which encompass approximately 68% of The Gambia’s population across urban, peri-urban and rural settings, a mapping exercise identified 42 practising TBS, of whom 33 (78.6%) participated. TBS reported suspected cases through WhatsApp groups and weekly follow-up calls, including cases described locally as ‘hip dislocations’. Combined with recruitment through participating healthcare facilities, of which 15 of 16 identified hospitals and diagnostic facilities in the study areas participated, including all hospitals in The Gambia capable of providing surgical hip fracture care, this framework aimed to capture the full spectrum of hip fracture care pathways within the study setting. Patient recruitment procedures started in October 2021 and were enhanced from July 2022 through additional research staff training and refinement of data collection tools. Data collection was prioritised for Edward Francis Small Teaching Hospital (EFSTH)—the tertiary referral hospital in Banjul, the capital city of The Gambia, to focus study resources on public healthcare. EFSTH is the only tertiary referral hospital in the country and the site of ~99% of all public hospital provided hip fracture surgeries.

Hip fractures were primarily confirmed through radiographic assessment and classified as intracapsular (ICD-10 code S72.0), pertrochanteric (ICD-10 code S72.1) and subtrochanteric (ICD-10 code S72.2). In cases where radiographs were unavailable—due to equipment failure, power outages or financial constraints—a structured clinical diagnosis protocol was employed independently by two orthopaedic surgeons using the mechanism of injury, patient symptoms and examination findings.^16^ Where there was disagreement, a third senior orthopaedic surgeon reviewed the case and a consensus reached.

Consenting patients were prospectively followed for 12 months using a structured, multimodal approach to ensure comprehensive data capture. Trained researchers recorded clinical outcomes, resource use and quality of life (EQ-5D-5L) at admission and at 1, 4 and 12-month follow-up through home visits, with telephone follow-up for remote participants.

### Resource use measurement and valuation

Healthcare resource use was identified and quantified for each patient’s specific care pathway during the clinical management of hip fracture expenditures (online supplemental figure S1). Patient care pathways were developed in consultation with local orthopaedic surgeons in The Gambia and encompass two main clinical strategies: operative and non-operative management (see online supplemental appendix for a detailed care pathway description). The non-operative pathway includes conservative medical care within the formal healthcare system (eg, skin/skeletal traction). Patients frequently utilised combined care pathways, seeking care from both TBS and formal biomedical healthcare facilities during their treatment journey. Consequently, baseline stratifications by facility type (public facility, private facility or TBS) reflect the site of primary study recruitment rather than mutually exclusive treatment pathways. These pathways, along with reviews of a sample of inpatient forms and hospital bills, informed the development of our data collection tools, which were subsequently validated with in-country orthopaedic specialists. Data were collected on resources used for care received before recruitment, healthcare facility/TBS visits (initial and subsequent), healthcare facility in-patient care (initial and subsequent admission), outpatient follow-up and any community-based care.

Healthcare resource use data were collected through multiple validated approaches: (1) systematic abstraction from patient records or hospital bills (in the case of private patients) using standardised case report forms; (2) structured interviewer-administered questionnaires with patients and caregivers and (3) consultations with treating orthopaedic specialists and clinic staff to verify resource utilisation. This triangulated approach ensured comprehensive capture of resource use across the entire care pathway.

Data on healthcare resource utilisation during healthcare facility visits and in-patient care (initial and subsequent admission) were primarily abstracted from patients’ medical records. Data included emergency/outpatient department visits, ward admission, theatre/operation, surgical implants, radiographs, investigations/blood tests, medications, consumables, blood products and walking aids.

Resources used for healthcare received before study enrolment and after initial healthcare facility/TBS presentation were collected using a bespoke researcher-administered questionnaire, completed with the patient and/or their caregiver. Data included outpatient healthcare facility visits, TBS visits, hospital readmissions, home visits by healthcare professionals, medications, home adaptations or modifications, specialist equipment costs and assistive devices (eg, walking aids). Resource use for patients who died before a given follow-up timepoint was treated as missing at that timepoint and recorded as zero for all subsequent timepoints.

Unit costs required to value resources used in the treatment journey for a hip fracture in The Gambia were not readily available and hence were derived from first principles using expenditure data from EFSTH finance department and the Ministry of Health in The Gambia (see details in online supplemental appendix). These unit costs were generated for: (1) emergency department visits; (2) surgical outpatient department visits; (3) surgery (taken as any type of surgery); and (4) the admission in a surgical ward (total stay). Although more granular data on resource use were obtained and reported, it was only possible to apply the unit costs created at a grouped level of resource expenditures (online supplemental table S2). For patients admitted to public hospitals, resources used during initial and subsequent in-hospital care were valued using these generated unit costs. Implants were valued using market-based unit costs estimated in consultation with the resident specialist orthopaedic surgeon. Resource use for patients admitted to private hospitals was valued using cost data provided with the medical bills. Resource use for care received before recruitment including TBS care, additional/outsourced resources used by patients during admission and postdischarge was valued using patient and/or caregiver (proxy when patients were unable to respond directly) reported costs. These included healthcare facility visits, TBS visits, medicines, home visits, home adaptations and equipment/walking aids. These data were collected using a bespoke data collection tool administered by trained research staff in accordance with a standardised operating procedure.

All costs were collected in local currency, the Gambian Dalasi (GMD). Costs in local currency units were first adjusted for inflation to 2024 prices using local consumer price indices obtained from the World Bank.^7^ The inflation-adjusted costs were then converted to International Dollars (I$) using the purchasing power parity conversion factor from the World Bank. This approach allows for cross-country comparisons by accounting for both local inflation and differences in purchasing power.

### Health outcome measurement and valuation

Quality-adjusted life years (QALYs) were used as the primary health outcome measure. QALYs were derived from patient-reported health status collected using the EuroQol 5-Dimension 5-Level (EQ-5D-5L) and EuroQol Visual Analogue Scale (EQ-VAS) instruments.^17^ Patients were asked to retrospectively report their health status immediately prior to their hip fracture at baseline and to describe their health on the day of follow-up.

Responses to the EQ-5D-5L questions were converted to utility scores using a value set for Ghana,^18^ as no country-specific value set is currently available for The Gambia and as a West African country represents the most similar data to apply. Patients who died before a given follow-up timepoint were assigned a utility score of 0 for that time point and all subsequent timepoints. QALYs for each patient were calculated using the area under the curve approach.^19^ This approach involves calculating the mean of the utility scores for two consecutive timepoints, multiplying it by the time interval (expressed in years), and summing across all time intervals to obtain total QALYs.

### Statistical analysis

#### Analysis of resource use and costs

For each resource category, the number of patients receiving a service, mean resource use and SD were calculated. These were stratified by surgical status (surgical vs non-surgical), based on observed data without imputation for missing values. Using this unimputed data, for each patient, total costs at each follow-up timepoint were estimated by summing costs across all healthcare resource items. Unadjusted mean total costs alongside the 95% CIs were calculated using bias-corrected and accelerated (BCa) bootstrap methods.

#### Analysis of health outcomes

Mean utility scores (with SD) were calculated for both the surgical and non-surgical cohorts at baseline and all follow-up assessments, using complete-case analysis where no imputation was performed for missing data. Total unadjusted mean QALYs per patient, along with corresponding BCa 95% CIs, were also estimated for each group.

#### Missing data

Missing data were assumed to be missing at random, based on the patterns of missingness established from a descriptive analysis, and were imputed based on observed data using multivariate imputation by chained equations (MICE) with predictive mean matching,^20^ stratified by surgical and non-surgical treatment. At each timepoint, costs were imputed separately for each cost category, while utility data were imputed as a single aggregate health state utility value rather than for each individual domain. The imputation models included age at admission, sex, ethnicity, residence (urban/rural), place of recruitment (public facility, private facility or TBS), presentation delay (defined as greater than 2 weeks after injury), history of previous fracture, fracture classification, 12-month follow-up status (alive, lost-to follow-up, dead) and observed outcomes (utility and costs) as predictors. Complete follow-up data were available for 62% of patients; thus, 40 complete datasets were generated as recommended.^21^ The imputation procedure was implemented using the *mice* R package.^20^

#### Economic evaluation

The imputed datasets were analysed using seemingly unrelated regressions via the *systemfit* package in R^22^ to estimate adjusted mean differences in total costs and QALYs between treatment groups. Two linear regression equations (one for costs, one for QALYs) were jointly modelled, allowing for the correlation between the error terms for these outcomes. The QALY regression adjusted for treatment assignment, age, sex, ethnicity, residence (urban/rural), place of recruitment (public facility, private facility or TBS), presentation delay (taken as 2 weeks after injury), history of previous fracture, fracture classification and baseline utility, while the cost regression included all these covariates except baseline utility. Parameter estimates obtained from each imputed dataset were pooled using Rubin’s rules^21^ and used to calculate the incremental cost-effectiveness ratios (ICERs) by dividing the difference in adjusted costs by the difference in adjusted QALYs. Statistical uncertainty was assessed using non-parametric bootstrapping with 1000 replicates to generate robust CIs around the cost and QALY estimates. Results are shown using a cost-effectiveness plane, plotting incremental costs against incremental QALYs and through cost-effectiveness acceptability curves that depict the probability of the intervention being cost-effective at varying thresholds. A health economic analysis plan was developed and is available on request. We complied with the Consolidated Health Economic Evaluation Reporting Standards (CHEERS 2022) reporting guidelines.^23^

#### Sensitivity analysis

To assess the robustness of our findings, we conducted a number of sensitivity analyses: (1) restricting the analysis to patients who had surgery within 3 days of admission (39%; 25/64) to assess impacts of timely care, consistent with the new AO (Arbeitsgemeinschaft für Osteosynthesefragen – Association for the Study of Internal Fixation) alliance guidelines for femur fracture^24^; (2) recalculated unit costs by reallocating overhead and intermediate costs to final cost centres using alternative allocation bases (personnel numbers) rather than direct expenditures (online supplemental table S1 and figure S2); (3) evaluated cross-country utility valuation effects by applying Zimbabwean EQ-5D crosswalk valueset^25^ instead of Ghanaian tariffs; (4) restricted the analyses to patients recruited in public facilities only (60%; 82/137) to isolate sector-specific practice patterns; (5) assessed missing data assumptions by using simple mean imputation for missing utilities, inflating values by 10% to correct for potential underestimation; (6) restricting the analysis time horizon to 120 days to control for potential data incompleteness following mid-study protocol revisions and (7) restricting the analysis to patients enrolled after July 2022 (72%; 99/137) to control for temporal improvements in data quality following mid-study protocol revisions. Finally, we additionally adjusted for baseline comorbidities (presence vs absence) and postinjury complications (presence vs absence) to confirm the robustness of our estimates.

### Ethical and governance approvals

Ethical and governance approvals were obtained from The Gambia Government/MRC Unit The Gambia@LSHTM Scientific Coordinating Committee and Ethics Committee (22 April 2021 ref 22975); Ministry of Health (20 August 2021 ref DDHS/AD/2021/08(MTN27))

### Patient and public involvement

Hip fracture patients experience severe pain, anxiety and distress following their injury, so it was not considered appropriate to seek their direct input for this observational study. Instead, we engaged a wide range of community-based and hospital-based healthcare providers to map the patient care pathway and ensure the study design maximised opportunities to identify all hip fracture cases.

## Results

### Study population

The cohort comprised 137 patients with hip fractures who provided informed consent and baseline data (online supplemental figures S3 and S4): 60% (n=82) female, mean age of 70 years (SD: 13.4) and 65.7% (n=90) living in urban areas (online supplemental table S3). Most patients (81.8%, n=112) were recruited through formal healthcare (59.9% public, 21.9% private) and 18.2% (n=25) via TBS. While 53% (n=73) were hospitalised, only 47% (n=64) underwent surgery. At 12 months, 79.6% (n=109) were alive and active in the study, 5.1% (n=7) were lost to follow-up and 15.3% (n=21) had died. Complete cost and QALY data across all four assessment timepoints were available for 62.0% (85/137) of patients, with higher completion among non-surgically managed patients (69.9% vs 53.1%; online supplemental table S4 and S5, online supplemental figures S5 and S6).

### Resource use and costs

Surgically managed patients had higher formal healthcare utilisation across all categories, whereas non-surgical patients reported more TBS visits (mean 5.53 (SD 5.91) vs 1.52 (SD 4.09); online supplemental table S6). Follow-up resource use was generally low (<30% reported). Unadjusted mean total costs (complete-case analysis) were substantially higher for surgery (US$1086 (95% CI $977 to $1201) vs US$143 (95% CI $96 to $200); table 1, online supplemental table S7 and figure S7). Surgical costs were driven by procedures (US$494; 45.5%), implants (US$319; 29.4%) and initial admissions (US$306; 28.2%). Conversely, non-surgical costs stemmed mainly from prerecruitment care (US$50; 35.0%), emergency department visits (US$44; 30.8%) and initial admissions (US$34; 23.8%).

**Table 1:** Mean unadjusted total costs aggregated over the main resource categories over the 12-month follow-up period

Resource	Operated		Not operated	
	n	Mean cost—US$ (95% CI)	n	Mean cost—US$ (95% CI)
Prior care	62	35 (6 to 78)	73	50 (30 to 76)
Emergency department attendance	63	83 (57 to 113)	73	44 (26 to 69)
Outpatient department attendance	63	5 (3 to 7)	73	3 (1 to 4)
Hospital admission	64	306 (175 to 474)	73	34 (13 to 68)
Surgery	64	494 (450 to 547)	73	0 (0 to 0)
Surgical implant	59	319 (287 to 355)	73	0 (0 to 0)
Follow-up hospital admission	60	6 (0 to 17)	71	0 (0 to 0)
Facility visit	59	23 (3 to 49)	70	2 (0 to 5)
Home visit	51	3 (0 to 6)	60	0 (0 to 0)
Home adaptation*	60	0 (0 to 0)	71	0 (0 to 0)
Equipment/walking aids	60	4 (2 to 7)	70	5 (2 to 8)
Medicines	53	3 (0 to 9)	67	2 (0 to 3)
Total cost	37	1086 (977 to 1201)	54	143 (96 to 200)

Mean costs were calculated using observed data without imputation for missing values (complete-case analysis). All costs are presented in 2024 US$.

* Home adaptation refers to any structural changes (eg, installation of railings) to the patient’s home to enable them to manage better after hip injury.

### Health state utility scores and QALYs

Prior to the hip fracture, most patients reported no EQ-5D-5L problems across all domains (66%–89%), indicating good baseline health (figure 1A). At 30 days, both groups experienced some degree of impairment, with only 6%–15% reporting no problems. Despite partial recovery by 120 days and 12 months, health status remained substantially below baseline. However, surgical patients showed superior recovery, being 1.5 times more likely to report no problems across all domains. Mean utility scores mirrored the same pattern; dropping sharply at 30 days before recovering, with surgical patients achieving higher utility at all timepoints except 30 days (figure 1B). EQ-VAS remained static (~75 on a 0–100 scale) throughout (online supplemental figure S8). Unadjusted mean QALYs (complete-case analysis) were 0.38 (95% CI 0.26 to 0.49) for surgery versus 0.27 (95% CI 0.16 to 0.37) for non-surgical care (see online supplemental figure S9 for distributions).

**Figure 1:**
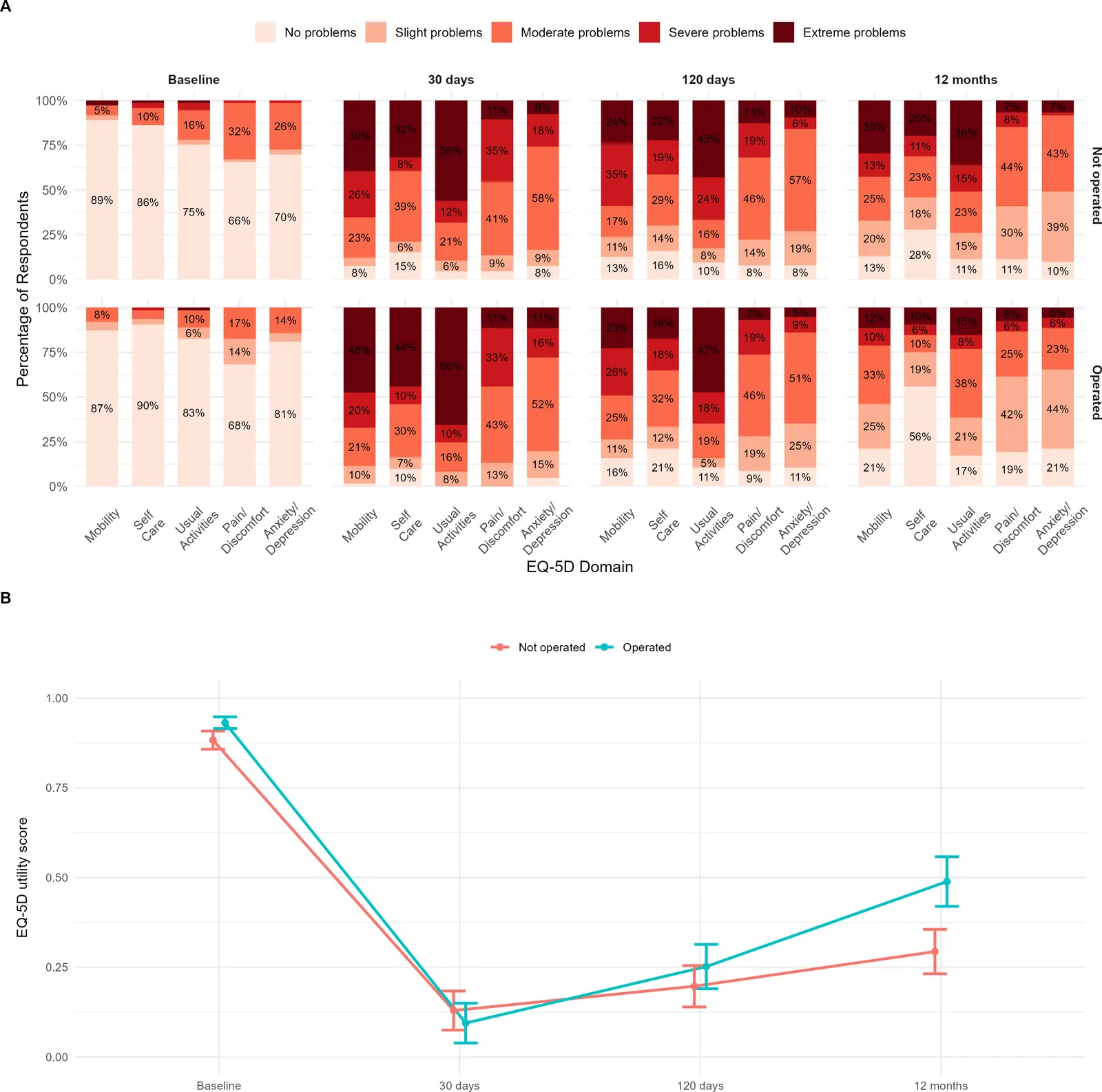
EQ-5D health states across domains (A) and unadjusted mean EQ-5D utility scores (B) at baseline and each timepoint over the 12-month follow-up period stratified by operation status. The mean EQ-5D utility scores were calculated using observed data without imputation for missing values (complete-case analysis). EQ-5D, European Quality of Life (EuroQol) 5 Dimensions.

### Economic evaluation

After covariate adjustment, patients who received surgery had, on average, US$988 (95% CI US$844 to US$1132) higher costs and gained 0.122 (95% CI −0.034 to 0.277) more QALYs compared with those who did not receive surgery (table 2). While the difference in costs was consistently higher, the estimated QALYs gained suggest some uncertainty regarding health gains, as indicated by the CI extending below zero (figure 2). This resulted in an ICER of US$8117 per QALY gained for surgery compared with no surgery. Based on the cost-effectiveness acceptability curve, the probability of surgery being cost-effective exceeded 50% at willingness-to-pay thresholds of US$8200 per QALY gained or higher (figure 3).

**Figure 2:**
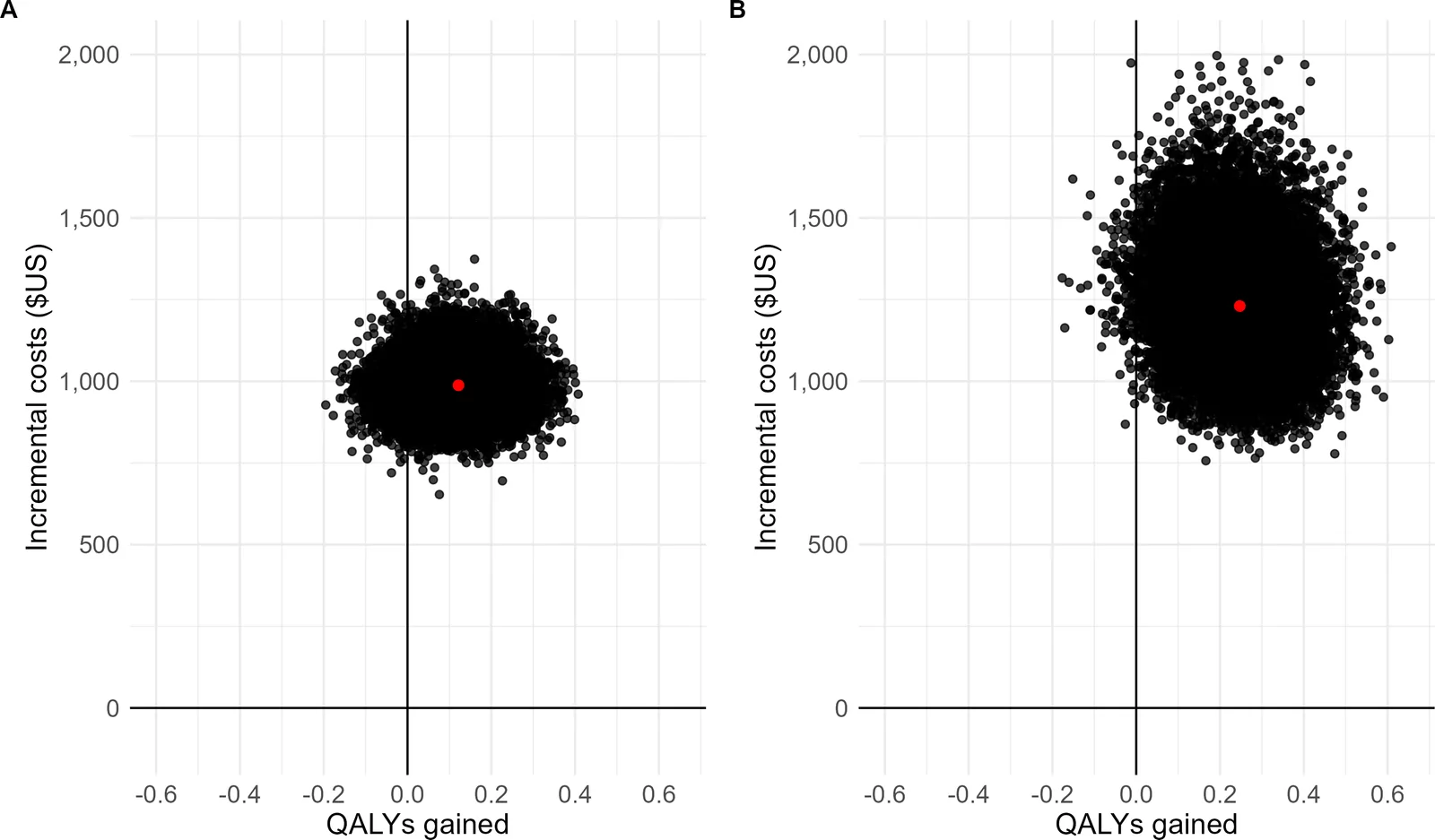
Cost-effectiveness planes showing the differences in costs (y-axis) and quality-adjusted life-years (QALYs) gained (x-axis) of surgical treatment of hip fracture, compared with non-surgical treatment based on 1000 bootstrap samples. (A) (left) shows base case results based on covariate adjusted regression and (B) (right) shows results when the analysis is restricted to patients who received surgery within 3 days of admission. The red dot represents the mean incremental costs and QALYs gained.

**Figure 3:**
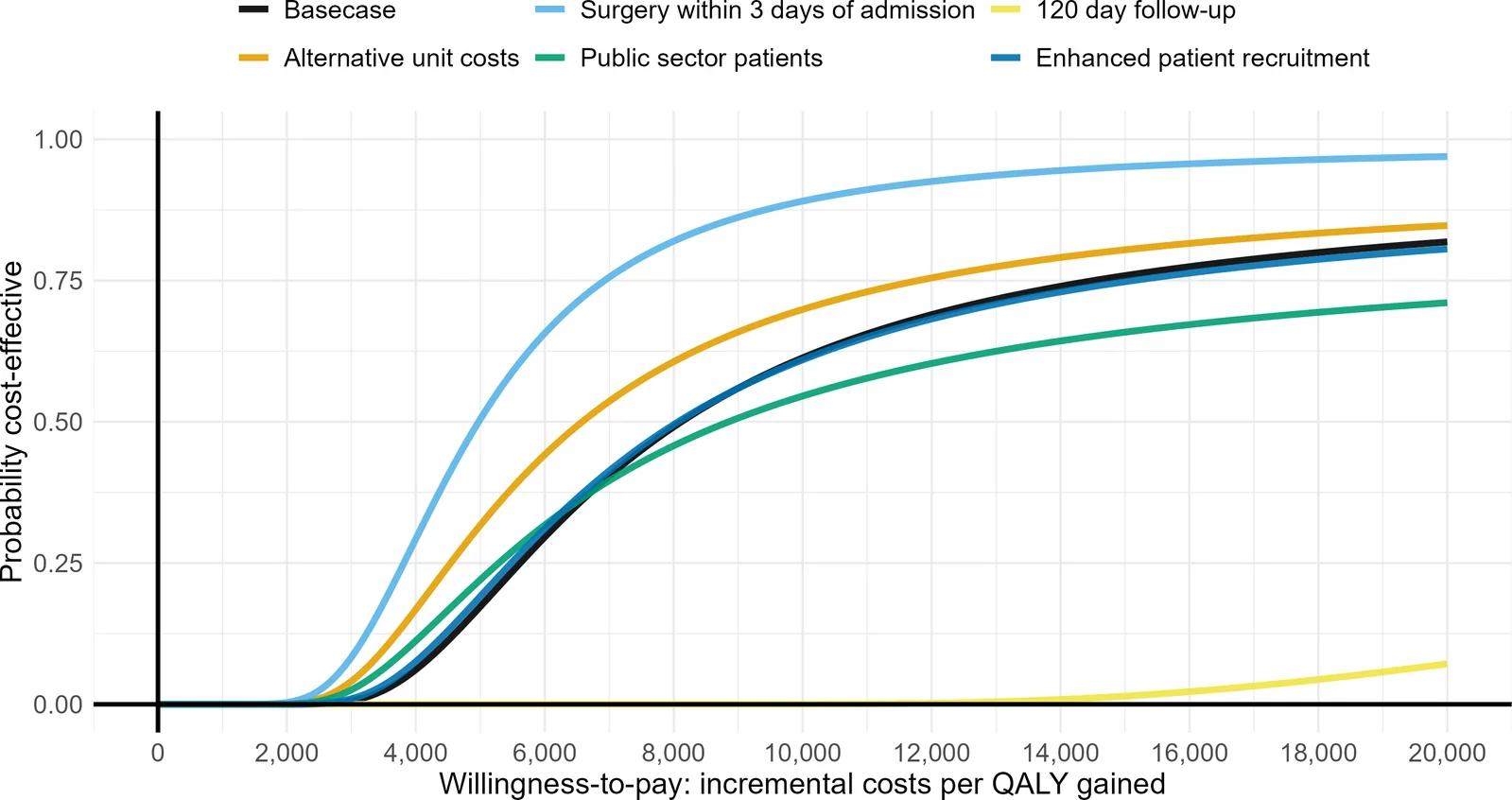
Cost-effectiveness acceptability curves showing the probability of surgical treatment being cost-effective (y-axis) compared with non-surgical treatment over a range of different decision-makers’ cost-effectiveness threshold choices (x-axis). Alternative unit costs—unit costs for facility visits, admissions and surgery were recalculated by reallocating overhead and intermediate costs to final cost centres using alternative allocation bases (personnel numbers) rather than direct expenditures. Enhanced patient recruitment—procedures were enhanced through additional training of research staff, modifications to the data capture tools and prioritising data collection at Edward Francis Small Teaching Hospital. All costs are presented in 2024 US$. QALYs, quality-adjusted life-years.

**Table 2:** Total adjusted QALYs, costs and cost-effectiveness analysis

	n	Total costs (95% CI)	QALYs (95% CI)	ICER (US$ per QALY gained)
Base case				
Operated	64	1198 (1054 to 1341)	0.379 (0.2651 to 0.493)	
Not operated	73	210 (130 to 291)	0.2573 (0.153 to 0.362)	
Difference		988 (843 to 1132)	0.1217 (–0.0341 to 0.2774)	8117
Patients who had surgery within 3 days of admission only				
Operated	25	1,407 (1,083, 1,732)	0.485 (0.32, 0.65)	
Not operated	73	210 (129, 291)	0.258 (0.157, 0.358)	
Difference		1,230 (908, 1,553)	0.247 (0.056, 0.439)	4,974
Public hospital patients only				
Operated	46	972 (926 to 1,017)	0.37 (0.24 to 0.5)	
Not operated	36	137 (79 to 194)	0.28 (0.14 to 0.41)	
Difference		835 (759 to 911)	0.094 (–0.09 to 0.28)	8,852
Additional covariates (pre-existing comorbidity status and post-injury complications)				
Operated	64	1203 (1055 to 1351)	0.385 (0.276 to 0.494)	
Not operated	73	206 (120 to 291)	0.252 (0.144 to 0.36)	
Difference		997 (839 to 1156)	0.133 (–0.021 to 0.288)	7489
Alternative unit costs*				
Operated	64	1028 (875 to 1181)	0.3789 (0.265 to 0.4929)	
Not operated	73	228 (142 to 315)	0.2574 (0.1526 to 0.3622)	
Difference		800 (644 to 955)	0.1215 (–0.0343 to 0.2773)	6580
120-day time horizon				
Operated	64	1200 (1055 to 1345)	0.0934 (0.0617 to 0.1251)	
Not operated	73	210 (129 to 291)	0.0765 (0.0478 to 0.1051)	
Difference		990 (843 to 1137)	0.017 (–0.0262 to 0.0602)	58 359
Simple mean imputation for utilities†				
Operated	64	1199 (1055 to 1342)	0.377 (0.267 to 0.488)	
Not operated	73	210 (129 to 291)	0.258 (0.157 to 0.358)	
Difference		989 (844 to 1133)	0.12 (–0.03 to 0.27)	8262
Zimbabwe EQ-5D-5L cross-walk value set‡				
Operated	64	1198 (1054 to 1341)	0.515 (0.4389, 0.5912)	
Not operated	73	210 (130 to 291)	0.443 (0.3731, 0.5129)	
Difference		988 (843 to 1132)	0.072 (–0.033 to 0.177)	13 717

All costs and QALYs were estimated using regression models adjusted for age, sex, ethnicity, residence (urban/rural), place of recruitment (public facility, private facility or TBS), history of previous fracture, fracture classification and baseline utility.

* Unit costs for facility visits, admissions and surgery were recalculated by reallocating overhead and intermediate costs to final cost centres using alternative allocation bases (personnel numbers) rather than direct expenditures.

† Missing data points were replaced with the mean of observed values, and the imputed values were inflated by 10% to account for potential underestimation.

‡ The EQ-5D-5L ‘crosswalk’ value set for Zimbabwe (instead of the Ghanaian EQ-5D-5L value set used in the base-case) was applied to health-related quality of life data to generate utility values used to calculate QALYs. All costs are presented in 2024 US$.

EQ-5D-5L, EuroQol 5-Dimension 5-Level; ICER, incremental cost-effectiveness ratio; QALYs, quality-adjusted life-years; TBS, traditional bone setters.

### Sensitivity analyses

Restricting the analysis to patients who received surgery within 3 days of admission (table 2) resulted in greater health benefits, showing larger QALYs gained (0.247 (95% CI 0.056 to 0.439)) compared with the full surgical group (0.122 (95% CI −0.034 to 0.277)). This translated into a more favourable cost-effectiveness profile, with a lower ICER of US$4974 per QALY gained for surgery versus no surgery. Sensitivity analyses including comorbidities and postinjury complications produced consistent cost and QALY estimates, reducing the ICER from US$8117 to US$7489 per QALY gained (table 2), largely due to a ~10% increase in incremental QALYs with surgery. Restricting the analysis to patients recruited from public healthcare facilities reduced overall costs and narrowed the cost difference between surgical and non-surgical groups. However, the health benefits of surgery were also smaller in this subset, with fewer QALYs gained. As a result, despite lower costs, the ICER increased to US$8852 per QALY gained—higher than in the primary analysis (table 2). Applying an alternative approach to estimating unit costs (allocating overhead and intermediate costs to final cost centres using personnel numbers instead of direct expenditures) reduced the total costs for patients who received surgery resulting in a smaller difference in costs (US$800 (95% CI $644 to $955)) compared with patients who did not receive surgery. This resulted in an ICER of US$6580/QALY gained. Calculating QALYs based on the EQ-5D-5L ‘crosswalk’ value set for Zimbabwe (instead of the Ghanaian EQ-5D-5L value set used in the base-case) resulted in a smaller difference in QALYs and a higher ICER of US$13 717/QALY gained. A smaller accumulation of QALYs and slower recovery for patients who received surgery resulted in a much smaller gain in QALYs and a higher ICER of US$58359/QALY gained when using a shorter (120 days) time horizon. The analysis restricted to patients who received surgery within 3 days of admission had higher probabilities of being cost-effective across a wide range of cost-effectiveness thresholds (figure 3).

## Discussion

Surgical management of hip fracture is more costly but led to clinically meaningful improvements in QALYs, with an average gain of 0.12 QALYs per patient, translating to approximately an extra 44 days in good health. This exceeds the range of suggested minimal clinically important difference of 0.050 to 0.075.^26^ This substantial gain underscores the significant value of surgical intervention in restoring meaningful function and quality of life to patients following a debilitating injury.

Care pathways for hip fracture in The Gambia are complex and fragmented, involving multiple stakeholders who often operate independently. This lack of coordination contributes to systemic inefficiencies in care that compromise patient outcomes. A WHO Service Availability and Readiness Assessment (SARA) survey in The Gambia identified critical gaps along the hip fracture care continuum, pointing to opportunities for system strengthening.^8^ This fragmentation in a complex care pathway in part accounted for our need to revise data collection processes during our study.

Our sensitivity analysis showed that patients who had surgery within 3 days gained twofold more QALYs than the overall surgical cohort. Improving patient care pathways, particularly by ensuring early presentation and timely surgery, could enhance patient outcomes and reduce costs. Delays in care were common in this cohort: 43% (59/137) of patients presented to a health facility more than 2 weeks after injury, and among those who received surgery, 39% (25/64) experienced operative delays exceeding 3 days postadmission. These delays, often stemming from the need for patients or families to secure funds for surgery (Touray *et al*, unpublished), frequently lead to complications that increase costs of care and diminish the benefits of treatment. These delays had measurable clinical and economic consequences: while our sensitivity analysis showed higher costs for patients receiving early surgery, the accompanying health outcome improvements were substantial. Our findings align with a substantial body of international evidence, demonstrating that prompt surgical management significantly improves functional recovery, reduces mortality and downstream health costs.^27–29^ In low and middle-income settings like The Gambia where prehospital delays and health facility bottlenecks frequently prolong time to surgery, reducing treatment delays represents a crucial leverage point to improve health-related quality of life, prevent long-term disability and mitigate downstream healthcare costs.

Differences in practice between public and private healthcare facilities create challenges for accurate economic evaluation. These arise from potential variations in protocols, resource use and pricing structures, which can distort comparisons and limit generalisability. To address this, we conducted a sensitivity analysis of public-sector patients, where care pathways and costs are more likely to be standardised. Although this reduced costs and costs variation and allowed a more internally valid comparison, the QALYs gained were smaller and uncertain.

A key strength of this study is the use of detailed patient-level data to capture resource use and costs across the care continuum, including TBS care provision. This prospective approach enabled tracking of downstream costs over time—critical for hip fracture, where recovery is often incomplete and costs typically continue to accumulate.^30^ This rigorous approach led to the generation of the first robust economic evidence of hip fracture burden specifically from a West African context, where health systems are notably pluralistic, encompassing a complex mix of public, private and informal healthcare providers. Although critical for planning healthcare services, such evidence is rarely available. By mapping real-world care pathways and resource use within this complex system, our findings offer critical insights for policymakers aiming to improve service delivery and allocate resources more efficiently.

Another significant strength is the collection of self-reported data on health-related quality of life at multiple timepoints. This enabled the calculation of QALYs, an important patient-reported outcome measure. QALYs not only account for the duration of life but also its quality, offering a holistic perspective on the potential benefits of an intervention. These patient-centric data provide invaluable insights into what matters to patients, and whether they perceive improvements in their health and well-being following different treatment interventions. This direct patient feedback is vital for assessing the real-world impact and value of healthcare services. This patient-centred dimension strengthens the relevance of our results for both clinical decision-making and policy development.

Despite these strengths, the study has some methodological limitations. First, national unit costs for health services were unavailable, so we estimated them using detailed data from a major referral hospital and Ministry of Health inputs. While transparent, this approach may not fully reflect variations across other facilities. Scenario analyses using alternative allocation bases for indirect costs demonstrated how sensitive total cost estimates are to these assumptions, highlighting the need for improved cost accounting in such settings. Second, costs for non-surgical management are likely underestimated. Most patients managed non-surgically were not hospitalised and instead relied on TBS services, with costs estimated based on patient-reported payments. However, these estimates are particularly challenging to quantify accurately due to the informal nature of TBS services, where payments often involve non-cash transactions or informal arrangements that may not be fully captured in patient recall.

Third, the absence of a Gambia-specific EQ-5D value set necessitated the use of Ghanaian population tariffs to calculate health state utilities. While this is a pragmatic approach given the geographical proximity, it introduces uncertainty, as health valuations may vary across contexts. This concern may be greater given that The Gambia is a low-income country, whereas Ghana is middle-income. To account for this, we conducted a sensitivity analysis using an alternative tariff from Zimbabwe, a low-middle income country. The results showed our QALY estimates were affected by this choice, underscoring the importance of developing country-specific value sets to support more accurate evaluations. Additionally, retrospectively assessed baseline EQ-5D scores may be subject to recall bias, particularly given that 43% of patients presented more than 2 weeks after injury. However, this remains the standard and only feasible approach for establishing prefracture quality of life in hip fracture.^31^

Fourth, the logistical challenges experienced during the study potentially affected data completeness and quality. In response to concerns about incomplete case capture and data quality, recruitment was restarted on 1 July 2022, and full data collection limited to patients attending EFSTH from 7 February 2023 onward. While a 12-month follow-up was achieved, it was condensed and focused on resource use without cost data. Costs at 12 months were estimated using previously collected unit costs, but intermediate data at 6–8 months were not collected. This likely led to underestimation of total costs, particularly for patients needing prolonged care or rehabilitation. However, evidence from our previous work suggests most costs accrue during the initial hospitalisation.^12^

We also conducted a sensitivity analysis using a restricted 120-day time horizon. While short-term costs were similar to those observed over 12 months, QALY gains were minimal during this initial period. This likely reflects the characteristically slow and incomplete recovery from hip fracture, with many patients never regaining their preinjury quality of life^30^ and requiring ongoing support and resources. Longer follow-up (12 months) revealed more pronounced QALY gains for surgical patients, suggesting that the benefits of surgery become increasingly apparent as recovery trajectories diverge. Although full recovery is rarely achieved regardless of treatment approach, our data indicate that surgical intervention demonstrates more favourable cost-effectiveness when evaluated over a longer horizon that captures these differential health gains.

Finally, our observational design entails potential residual confounding and selection bias. Out-of-pocket costs may favour wealthier, healthier surgical candidates and non-response from some traditional bonesetters (9/42) and facilities (1/16) could affect representation. Although primary models adjusted for baseline EQ-5D, demographics, fracture characteristics and care pathways, residual bias remains possible. However, expanding covariate adjustment in sensitivity analyses to account for broader health status and clinical progression yielded consistent cost-effectiveness conclusions.

As in many low and middle-income countries, The Gambia has no formally established national cost-effectiveness threshold. The historical 1–3×GDP per capita benchmark used in a few local health-economic studies has faced widespread criticism for failing to reflect true health opportunity costs.^32 33^ To our knowledge, no formal health-economic evaluations assessing surgical procedures or acute trauma have ever been published for The Gambia. We present cost-effectiveness acceptability curves, which enable policymakers to evaluate the probability of cost-effectiveness across a range of preferred willingness-to-pay thresholds. Our primary analysis suggests that thresholds above approximately US$8200 increasingly favour surgical treatment over conservative care.

Although operative hip fracture management requires a higher resource commitment, our findings highlight critical policy priorities for The Gambia. First, subsidising orthopaedic care through the expanding NHIS could reduce catastrophic out-of-pocket costs and improve surgical access. Second, centralised national procurement of essential trauma implants could further lower costs and improve public spending efficiency. Finally, standardising referral pathways between primary care, traditional bonesetters and tertiary facilities, in line with AO Alliance guidelines,^24^ could also reduce prehospital delays and duplicate diagnostic costs.

Overall, this study provides novel evidence on the health outcomes, costs and cost-effectiveness of hip fracture care in a low-resource West African setting. Surgical treatment produced meaningful quality-of-life improvements beyond recognised clinical benchmarks. Crucially, these findings offer timely empirical evidence to support implementation of the new Gambia National Trauma and Injury Plan,^34^ guide investment in surgical capacity, referral systems and equitable trauma care as osteoporosis and fragility fractures increase across sub-Saharan Africa.

## Supplementary material

10.1136/bmjgh-2025-022219 online supplemental file 1

10.1136/bmjgh-2025-022219 online supplemental file 2

## Data Availability

Data are available upon reasonable request.
