# Supplementary material for: Health economic evaluation of hip fracture care pathways in The Gambia
Source: BMJ Glob Health. 2026 Sep 30;11(9):e022219. doi: 10.1136/bmjgh-2025-022219 (PMC13629887; doi:10.1136/bmjgh-2025-022219)
Supplement: Supplementary file 1 [file bmjgh-11-9-s001.docx]

**REFLEXIVITY STATMENT**

1. **How does this study address local research and policy priorities?**

The Government of The Gambia are committed to the principals of Universal Health Coverage and introduced a National Health Insurance Scheme (NHIS) in 2021. This research aligns with the goals of The Gambian National Health Insurance Scheme and provides evidence to inform decisions on service prioritization for expansion and coverage.

2. **How were local researchers involved in study design?**

KSM, AT, MKJ, and BT were involved in questionnaire design, decisions regarding choice of study sites, community and stakeholder engagement, and securing permissions from hospitals. The team ensured contextual relevance and language translation of materials. The team piloted study tools, processes and questionnaires, collected and cleaned data, contributed to interpretation and write up of findings.

3. **How has funding been used to support the local research team?**

Funding has provided secure employment for the team and administrative support staff since 2021, a well-equipped office and a dynamic research environment in which to work with regular team meetings; project-related travel expenses have been renumerated.

4. **How are research staff who conducted data collection acknowledged?**

KSM, MKJ, BT, and AT, orthopaedic surgeon, study co-ordinator, orthopaedic surgeon assistant, and research assistant (PhD Candidate) respectively, facilitated data collection, cleaning and analysis. They are all co-authors. All individuals and institutions who collected data or supported the data collection process are all thanked by name in the acknowledgments section of the paper.

5. **Do all members of the research partnership have access to study data?**

All non-anonymised data (consent forms) are held with the team in The Gambia and are not accessible to anyone outside of The Gambia. Anonymised data are held within a secure REDCap database accessible to both The Gambia and United Kingdom team members.

6. **How were data used to develop analytical skills within the partnership?**

Across the wider Fractures-E3 research programme (which includes colleagues in South Africa and Zimbabwe) we hold monthly Early Career Researcher training seminars for example, including, programming R (utilising data collected in Fractures-E3 projects), Critical Appraisal, Bias and Confounding, Referencing, to name a few. In addition, we hold 4-6 weekly project management group meetings where we review new results and discuss these together.

7. **How have research partners collaborated in interpreting study data?**

We have held monthly, then 6-weekly project management group meetings, plus annual week-long investigator meetings since 2020. Emerging data have been sequentially discussed as a team. NM analysed the data. Co-authors contributed to data interpretation and contextualisation.

8. **How were research partners supported to develop writing skills?**

The senior academics on the team supported the early career researchers by giving multiple rounds of feedback on the writing.

9. **How will research products be shared to address local needs?**

These data were presented to The Gambian Ministry of Health, and key stakeholders (including Traditional Bone Setters who play a role in the care of patients) and health-related NGOs during workshops held in The Gambia from 20-26 November 2024, following which a briefing report was sent to all. Local television covered this event. Findings will be disseminated to the public and private hospitals from which most data were collected.

10. **How is the leadership, contribution and ownership of this work by LMIC researchers recognised within the authorship?**

NM is an ECR and first author. MKJ is the study co-ordinator in The Gambia. HW and TM are the data managers in the UK. AB is the project manager and epidemiologist. KW and CG are the principal investigators in The Gambia and the UK. SMN is health economics work package (WP3) lead. Other co-authors include KSM (The Gambian orthopaedic surgeon), BT (The Gambian orthopaedic surgeon assistant), LG (WP1 Deputy) and AT (The Gambian research assistant and PhD student).

11. **How have early career researchers across the partnership been included within the authorship team?**

NM, HW, AB, MKJ, AT, BT and TM are all early career researchers. NM is the lead author. AT, MKJ, BT are from The Gambia and NM, HW, AB, and TM are from United Kingdom.

12. **How has gender balance been addressed within the authorship?**

There are seven men (NM, KSM, MKJ, TM, SMG, JM and MLC) and eight women (AB, AT, LG, BT, HW, KAW, CLG and SMN).

13. **How has the project contributed to training of LMIC researchers?**

AT is supported in a PhD which is ongoing. MJ is supported on a Masters which is ongoing. TM recently completed his PhD. An online early career researcher training programme has run since 2021 as part of this research programme (see (6)) - NM attended these sessions to improve his data analytical and interpretation skills.

14. **How has the project contributed to improvements in local infrastructure?**

Musculoskeletal research is now a core research theme at the MRC Unit The Gambia at LSHTM; invaluable expertise and field experience have been developed, and the reputation of the Unit has benefited from this investment. The project has established sustainable bi-directional relationships with older people in communities who have been empowered to take charge of their health and wellbeing.

15. **What safeguarding procedures were used to protect local study participants and researchers?**

During project set-up we wrote a project-specific safeguarding policy. Staff training and re-training included safeguarding of staff (e.g. avoidance of lone working in communities, seat belt policy) and of study participants (e.g. intervening on humanitarian grounds when needed). Risk reporting and safeguarding were standing agenda items on 4-6 weekly project management group meetings.
