# Supplementary material for: Health economic evaluation of hip fracture care pathways in The Gambia
Source: BMJ Glob Health. 2026 Sep 30;11(9):e022219. doi: 10.1136/bmjgh-2025-022219 (PMC13629887; doi:10.1136/bmjgh-2025-022219)
Supplement: Supplementary file 2 [file bmjgh-11-9-s002.docx]

Health economic evaluation of hip fracture care pathways in The Gambia

Nyashadzaishe Mafirakureva^1,2^, Kebba S Marenah^3^, Anya Burton^2^, Tafadzwa Madanhire^2^, Hannah Wilson^2^, Momodou K Jallow^4^, Awa Touray^4,6^, Lucy Gates^6^, Bintou Trawally^3^, Simon Matthew Graham^5^, James Masters^5^, Matthew L Costa^5^, Kate A Ward^4,7^, Celia L. Gregson^2,8^, Sian M. Noble^9^

1. Sheffield Centre for Health and Related Research, School of Medicine and Population Health, University of Sheffield, Sheffield, United Kingdom.
2. Musculoskeletal Research Unit, Translational Health Sciences, Bristol Medical School, University of Bristol, Bristol, United Kingdom.
3. Department of Orthopaedics & Trauma, Edward Francis Small Teaching Hospital, Banjul, The Gambia.
4. Medical Research Council Unit The Gambia, London School of Hygiene and Tropical Medicine, Banjul, The Gambia.
5. Oxford Trauma and Emergency Care, Nuffield Department of Orthopaedics, Rheumatology and Musculoskeletal Science, University of Oxford, Oxford, United Kingdom.
6. School of Healthcare Innovation & Enterprise, Faculty of Medicine, University of Southampton, Southampton, England, UK
7. Medical Research Council Lifecourse Epidemiology Centre, Human Development and Health, School of Healthcare Innovation & Enterprise, Faculty of Medicine, University of Southampton, Southampton, England, UK
8. The Health Research Unit Zimbabwe, Biomedical Research and Training Institute, Harare, Harare Province, Zimbabwe
9. Population Health Sciences, Bristol Medical School, University of Bristol, Bristol, United Kingdom.

# Additional methods

## Patient care pathways

The clinical management of hip fractures in The Gambia is complex due to the pluralistic nature of the healthcare system. An illustrative care pathway was developed in consultation with the study teams and an in-country specialist orthopaedic surgeon (Touray et al., unpublished). Following injury, patients may present either to Traditional Bone Setters (TBS) or to a healthcare facility (public/private). Those who present to TBS may be managed exclusively there or subsequently transfer to a healthcare facility, often after considerable delay. Conversely, patients who initially seek care at a healthcare facility may later visit a TBS. As a result, patients frequently move back and forth between these two sectors of care. This dynamic "cycling" between traditional and formal healthcare providers is a key feature of the care pathway. The care pathway for patients presenting to healthcare facilities describes here is a typical patient’s treatment journey for a hip fracture at Edward Francis Small Teaching Hospital (EFSTH), the country’s only major referral public hospital. Typically, the patient with a suspected hip fracture presents to the Accident & Emergency (A & E) department where they are registered into the system upon payment of an A & E registration fee. The patient undergoes an initial assessment which typically includes blood tests such as haemoglobin (Hb) and blood grouping, with additional tests like full blood count (FBC), renal function, and urea and electrolytes (U&Es), often completed at an external facility if not available on-site. An X-ray is standard unless it has been previously conducted at another facility. It may be referred out due to non-functional in-house equipment. Pain management is also done and usually begins with analgesics such as diclofenac and tramadol.

Pre-operative care may include further investigations, although not uniformly done for all patients. These may involve an electrocardiogram (ECG), echocardiogram (ECHO), and a chest X-ray. Pre-operative prescriptions often include an orthopaedic implant, a standard theatre pack (comprising normal saline x3, iodine, ceftriaxone x2, and IV paracetamol x2), with possible additions like tranexamic acid, and two blood collections. These prescriptions are usually dispensed outside of EFSTH/Ndemban - with patients paying out-of-pocket. Patients may be managed non-surgically (either by clinician decision or patient preference) through conservative approaches, either within the hospital or in the community. In the latter case, they may seek care at a TBS clinic. Following surgery, care involves a post-op X-ray, follow-up Hb testing, continued analgesic administration, and physiotherapy. Upon discharge, the patient may face a combination of inpatient stay costs and the overall operation cost. In some cases, patients may need to purchase mobility aids independently if these are not provided by the hospital. Transportation home is typically via taxi, and additional outpatient physiotherapy may be required to support recovery.

**Figure S1.** Typical operative care pathway for hip fractures in The Gambia. A & E = accident and emergency, CXR=chest x-ray, ECHO= echocardiogram, ECG= electrocardiogram, FBC=full blood count, HB= haemoglobin, TXA=Tranexamic acid, U&E=urea and electrolytes, TBS=Traditional Bone Setter.

## Unit cost analysis

There is a paucity of unit costs required to value resources used in the treatment journey for a hip fracture in The Gambia and this necessitated calculation of unit costs from first principles using line-item expenditure data and guidance provided in Shephard et al.^1^ The process involves allocating expenditure to cost centres in order to calculate unit costs and follows the following steps:

1) Define the final product
2) Define cost centres
3) Identify the full cost for each input
4) Assign inputs to cost centres
5) Allocate all costs to final cost centres (compute total allocated costs)
6) Compute unit cost for each final cost centre

### The final product

The aim of this unit cost analysis was to calculate unit costs required to estimate the monetary value of resources utilised by patients during their treatment journey for a hip fracture. These unit costs were generated for: (1) emergency department presentation (A & E surgical Triage); (2) surgical outpatient department visit; (3) surgery (any type of surgery); and (4) admission in a surgical ward (total stay). Although ideal for our analysis, estimating the unit cost for orthopaedic ward stay and an orthopaedic surgery was not feasible.

### Cost centres

The main cost centres identified from the hospital’s financial records and used as the basis for the analysis include:

1. Overheads - provide overhead support to all cost centres
   a. Utilities
   b. Administration & accounts
   c. Security, works and maintenance
   d. Domestic service unit
   e. Other overheads
2. Clinical Support Services - provide service support to patient care cost centres
   a. Catering
   b. Laundry
   c. Equipment including oxygen
   d. Medical records
   e. Drugs and consumables
   f. Central Sterile Supplies Unit (CSSU)
3. Intermediate Departments - provide services to support patient care cost centres
   a. Lab medicine
   b. Radiology
   c. Physiotherapy
   d. Nursing
   e. Overseas travel and training clinical
   f. TAC(Technical Assistance expatriate consultants)
   g. Surgery
4. Patient Care - provide direct patient services
   a. Theatre
   b. A & E (surgical and intensive care unit)
   c. Surgical inpatient wards
   d. Surgical outpatient clinics
   e. Other patient centres

### Full cost for each input

The costs of inputs used in running the hospital were obtained from the 2023 EFSTH expenditure data provided by the hospital’s finance department. This was supplemented with information on expenditures directly paid for by the Ministry of Health such as utilities, technical assistant expatriate salaries, and drugs and supplies from the Central Medical Store. To identify the full cost for each input, individual line items were reviewed, and similar items were identified and grouped together.

### Assigning inputs to cost centres

Itemized costs for inputs were carefully assigned directly or proportionately to the relevant cost centres: Overheads, Intermediate Departments and Clinical Support Services.

### Allocating costs to final cost centres

Costs assigned to Overheads, Intermediate Departments and Clinical Support Services cost centres were then allocated to Patient Care which represented our final cost centres. A step-down allocation approach (illustration provided in Figure S1) where overhead, intermediate and clinical support services costs were sequentially assigned from one cost centre to another until they reached the final cost centres. The different allocation basis (criteria or measures used to distribute overhead, intermediate and clinical support service costs to final cost centres) used in the analysis are summarised in Table S1. Alternative allocation bases were evaluated in a sensitivity analysis where direct expenditures were used to allocate costs for other overhead, utilities, security/works & maintenance and equipment (see Table S1).

### Compute unit cost

Unit costs for each final cost centre were calculated by dividing the total allocated cost with relevant utilisation data (service volume) obtained from EFSTH.

**Table S1.** Assumptions used to allocate overhead costs to different cost centres. A & E = accident and emergency, DSU=Domestic service unit, TAC=Technical Assistant expatriate consultants

| **Cost item** | **Allocation basis 1** | **Allocation basis 2** |
| --- | --- | --- |
| Utilities | Personnel numbers | Direct expense |
| Administration & accounts | Personnel numbers | Personnel numbers |
| Security & works & maintenance | personnel numbers | Direct expense |
| Domestic services unit | % of DSU personnel | % of DSU personnel |
| Other overhead | % of personnel | Direct expense |
| Catering | Number of admissions | Number of admissions |
| Laundry | Bed capacity | Bed capacity |
| Equipment: including oxygen gas | % of personnel | Direct expense |
| Medical records | % of direct expense for following areas, nursing, TAC, surgery, theatre, other patient centres | % of direct expense for following areas, nursing, TAC, surgery, theatre, other patient centres |
| Drugs and consumables | % of direct expense for all remaining areas (exclusion overseas travel and training, A & E) | % of direct expense for all remaining areas (exclusion overseas travel and training, A & E) |
| CSSU | % of direct expense to depts that use sterile supplies (exclusion physio, radiology, overseas travel, A & E) | % of direct expense to depts that use sterile supplies (exclusion physio, radiology, overseas travel, A & El) |
| Lab medicine | % of direct expense to depts use lab medicine (exclusion physio, radiology, overseas travel, A & E) | % of direct expense to depts use lab medicine (exclusion physio, radiology, overseas travel, A & E) |
| Radiology | % of direct expense to depts use radiology (exclusion overseas travel, A & E) | % of direct expense to depts use radiology (exclusion overseas travel, A & E) |
| Physiotherapy | % of direct expense to depts use physiotherapy (exclusion overseas travel, A & E) | % of direct expense to depts use physiotherapy (exclusion overseas travel, A & E) |
| Nursing | % of nurses in each dept (surgical dept allocated directly to surgical outpatients and wards). Theatre nurses split between those theatres covered by the surgical dept (6 theatres), and those that aren’t, (3 theatres). | % of nurses in each dept (surgical dept allocated directly to surgical outpatients and wards). Theatre nurses split between those theatres covered by the surgical dept (6 theatres), and those that aren’t, (3 theatres). |
| Overseas training | % of direct expense to surgery, theatre and other patient centre | % of direct expense to surgery, theatre and other patient centre |
| TAC | % of direct expenses to surgery, theatre and other patient centres. | % of direct expenses to surgery, theatre and other patient centres. |
| Surgery | % of staff time allocated to Theatre (6 theatres: doesn’t include maternityx2 or eyex1) A & E, surgical wards and outpatients | % of staff time allocated to Theatre (6 theatres: doesn’t include maternityx2 or eyex1) A & E, surgical wards and outpatients |

**Figure S2.** Illustration of the step-down allocation approach where overhead, intermediate and clinical support services costs were sequentially assigned from one cost centre to another until they reach the final cost centres. DSU= Domestic service unit

Table S2. Allocated total costs, service volume, and estimated unit costs. A & E=accident and emergency, GMD=Gambian Dalasi, £= Pound sterling, SA=sensitivity analysis.

| **Cost centre** | **Total Cost (GMD)** | **Volume** | **Units** | **Unit cost (GMD)** | **Unit cost (£) SA** | **Unit cost (£)** |
| --- | --- | --- | --- | --- | --- | --- |
| Theatre | 94758984 | 2313 | Number of operations | 40968 | 266 | 532 |
| A & E | 29394540 | 10332 | Number of attendances | 2845 | 48 | 37 |
| Surgical wards | 21186198 | 2642 | Number of admissions | 8019 | 124 | 104 |
| Surgical outpatients | 16079315 | 8267 | Number of visits | 1945 | 28 | 25 |

# Supplementary results

**Figure S3.** Flow diagram showing enrolment and follow-up of the hip fracture cohort (n = 187). LFTU=loss to follow-up

Table S3. Baseline characteristics of the hip fracture cohort, stratified by receipt of operation.

| **Characteristic** | **Overall (n=137)** | **Operated (n=64)** | **Not operated (n=73)** |
| --- | --- | --- | --- |
| Age, mean (SD) | 69.8 (13.4) | 70.3 (13.3) | 69.3 (13.6) |
| Female (%) | 82 (59.9) | 35 (54.7) | 47 (64.4) |
| Urban residence, n (%) | 90 (65.7) | 49 (76.6) | 41 (56.2) |
| Recruitment location, n (%) | | | |
| Public facility | 82 (59.9) | 46 (71.9) | 36 (49.3) |
| Private facility | 30 (21.9) | 18 (28.1) | 12 (16.4) |
| Traditional bone setter (TBS) | 25 (18.2) | 0 (0.0) | 25 (34.2) |
| Previous fracture, n (%) | 9 (6.6) | 6 (9.4) | 3 (4.1) |
| *Recruitment reset, n (%) | 99 (72.3) | 47 (73.4) | 52 (71.2) |
| Presentation delayed by >2 weeks from injury, n (%) | 59 (43.1) | 22 (34.4) | 37 (50.7) |
| Type of fracture, n (%) | | | |
| Intertrochanteric | 44 (35.2) | 23 (35.9) | 21 (34.4) |
| Intracapsular | 68 (54.4) | 34 (53.1) | 34 (55.7) |
| Subtrochanteric | 13 (10.4) | 7 (10.9) | 6 (9.8) |
| Had surgery within 3 days of admission, n (%) | 25 (39.0) | 25 (39.0) | 0 (0.0) |
| End of 12-month follow-up, n (%) | | | |
| Alive | 109 (79.6) | 51 (79.7) | 58 (79.5) |
| LTFU | 7 (5.1) | 4 (6.2) | 3 (4.1) |
| Dead | 21 (15.3) | 9 (14.1) | 12 (16.4) |
| *Recruitment after reset (enhancement) due to concerns about incomplete case capture and poor data quality. LTFU= lost-to follow-up | | | |

Table S4. Descriptive summary of the data.

| **Variable** | **N** | **Missing** | **Missing percent** | **Mean/%** | **Standard Deviation** |
| --- | --- | --- | --- | --- | --- |
| Age (years) | 137 | 0 | 0 | 69.8 | 13.4 |
| Sex (Male) | 137 | 0 | 0 | 40.2* |  |
| Ethnicity (Mandinka) | 137 | 0 | 0 | 57.7* |  |
| Residence (Rural) | 137 | 0 | 0 | 34.3* |  |
| Hospital presentation (Public) | 137 | 0 | 0 | 59.9* |  |
| Previous fracture | 137 | 0 | 0 | 6.6* |  |
| Recruitment after reset | 137 | 0 | 0 | 72.3* |  |
| Presentation delayed by >2 weeks from injury | 137 | 0 | 0 | 43.1* |  |
| Type of fracture (Intracapsular) | 125 | 12 | 8.76 | 54.4* |  |
| Had surgery within 7 days of admission | 64 | 0 | 0 | 40.6* |  |
| Vital status | 137 | 0 | 0 | 79.6* |  |
| Baseline utility | 136 | 1 | 0.73 | 0.91 | 0.91 |
| Utility at 1 month | 133 | 4 | 2.92 | 0.11 | 0.11 |
| Utility at 4 months | 133 | 4 | 2.92 | 0.22 | 0.22 |
| Utility at 12 months | 128 | 9 | 6.57 | 0.38 | 0.38 |
| Prior care | 135 | 2 | 1.46 | 43.21 | 43.21 |
| Emergency Department visit | 136 | 1 | 0.73 | 69.37 | 69.37 |
| Outpatient visit | 136 | 1 | 0.73 | 4.19 | 4.19 |
| Initial admission | 137 | 0 | 0 | 177.05 | 177.05 |
| Surgery | 137 | 0 | 0 | 137.64 | 137.64 |
| Surgical implant | 132 | 5 | 3.65 | 142.64 | 142.64 |
| Admission at 1 month | 133 | 4 | 2.92 | 0.31 | 0.31 |
| Facility visits at 1 month | 133 | 4 | 2.92 | 3.79 | 3.79 |
| Medicines at 1 month | 132 | 5 | 3.65 | 3 | 3 |
| Home visits at 1 month | 124 | 13 | 9.49 | 0.33 | 0.33 |
| Home adaptation at 1 month | 133 | 4 | 2.92 | 0 | 0 |
| Equipment/walking aids at 1 month | 133 | 4 | 2.92 | 3.26 | 3.26 |
| Admission at 4 months | 134 | 3 | 2.19 | 2.2 | 2.2 |
| Facility visits at 4 months | 134 | 3 | 2.19 | 7.48 | 7.48 |
| Medicines at 4 months | 134 | 3 | 2.19 | 0.34 | 0.34 |
| Home visit at 4 months | 129 | 8 | 5.84 | 0.69 | 0.69 |
| Home adaptation at 4 months | 134 | 3 | 2.19 | 0 | 0 |
| Equipment/walking aids at 4 months | 134 | 3 | 2.19 | 1.04 | 1.04 |
| Admission at 12 months | 133 | 4 | 2.92 | 0.01 | 0.01 |
| Facility visits 12 months | 131 | 6 | 4.38 | 0.01 | 0.01 |
| Medicines 12 months | 123 | 14 | 10.22 | 0.09 | 0.09 |
| Home visit 12 months | 126 | 11 | 8.03 | 0.01 | 0.01 |
| Home adaptation 12 months | 133 | 4 | 2.92 | 0 | 0 |
| Equipment/walking aids 12 months | 132 | 5 | 3.65 | 0.11 | 0.11 |
| *Represent proportion | | | | | |

**Figure S4.** Sankey diagram showing patient flow from baseline to the end of 12-months follow-up.

Table S5. The number and proportion of individuals with complete economic data by treatment group at each data collection time point.

| **Timepoint** | **All patients (n = 137)** | **Non-operated (n = 73)** | **Operated (n = 64)** |
| --- | --- | --- | --- |
| *All economic data (Costs & QALYs)* | | | |
| Baseline | 129 (94.2%) | 73 (100%) | 56 (87.5%) |
| 30 days | 123 (89.8%) | 65 (89%) | 58 (90.6%) |
| 120 days | 128 (93.4%) | 68 (93.2%) | 60 (93.8%) |
| 12 months | 109 (79.6%) | 60 (82.2%) | 49 (76.6%) |
| All timepoints | 85 (62%) | 51 (69.9%) | 34 (53.1%) |
| *Cost data only* | | | |
| Baseline | 129 (94.2%) | 73 (100%) | 56 (87.5%) |
| 30 days | 123 (89.8%) | 65 (89%) | 58 (90.6%) |
| 120 days | 129 (94.2%) | 69 (94.5%) | 60 (93.8%) |
| 12 months | 114 (83.2%) | 62 (84.9%) | 52 (81.2%) |
| All timepoints | 91 (66.4%) | 54 (74%) | 37 (57.8%) |
| *QALY data only* | | | |
| Baseline | 136 (99.3%) | 73 (100%) | 63 (98.4%) |
| 30 days | 133 (97.1%) | 71 (97.3%) | 62 (96.9%) |
| 120 days | 133 (97.1%) | 70 (95.9%) | 63 (98.4%) |
| 12 months | 128 (93.4%) | 70 (95.9%) | 58 (90.6%) |
| All timepoints | 125 (91.2%) | 68 (93.2%) | 57 (89.1%) |

**Figure S5.** Pattern of missing data on costs at baseline and follow-up time points. The plot suggests a mixed missingness pattern where some individuals have intermittent missing data (i.e missing data earlier time points but have data on subsequent timepoints while others have no intermittent missing data suggesting LTFU, withdrawal, or death.

**Figure S6.** Pattern of missing data on EQ-5D at baseline and follow-up time points. The plot suggests a mixed missingness pattern where some individuals have intermittent missing data (i.e missing data from earlier time points but have data on subsequent timepoints while others have no intermittent missing data suggesting LTFU, withdrawal, or death.

## Resource use and costs

Table S6. Mean resource use by receipt of operation for hip fracture

|  |  | **Operated (n = 64)** | | | **Not operated (n = 73)** | | |
| --- | --- | --- | --- | --- | --- | --- | --- |
| **Resource name** | **Resource unit (Number of)** | ***Patients with data, n (%)*** | ***Patients receiving service, n (%)^ϕ^*** | ***Resource use, mean (SD)*** | ***Patients with data, n (%)*** | ***Patients receiving service, n (%)^ϕ^*** | ***Resource use, mean (SD)*** |
| ***Care before coming to the hospital*** | | | | | | | |
| Prior care (healthcare facility visits) | Visits | 63 (98.4%) | 18 (28.1%) | 0.30 (0.50) | 73 (100%) | 14 (19.2%) | 0.25 (0.55) |
| Prior care (traditional bone setter visits) | Visits | 63 (98.4%) | 11 (17.2%) | 0.38 (1.02) | 73 (100%) | 46 (63%) | 1.38 (1.48) |
| ***Initial healthcare facility presentation*** | | | | | | | |
| Healthcare facility visit | Visits | 63 (98.4%) | 60 (93.8%) | 1.1 (0.5) | 73 (100%) | 50 (68.5%) | 0.7 (0.5) |
| Healthcare facility visit investigations | Investigations | 64 (100%) | 60 (93.8%) | 3.3 (2.33) | 73 (100%) | 50 (68.5%) | 1.04 (1.16) |
| Healthcare facility visit medicines | Medicines | 64 (100%) | 60 (93.8%) | 2.66 (2.74) | 73 (100%) | 50 (68.5%) | 0.77 (1.34) |
| Healthcare facility visit consumables | Consumables | 64 (100%) | 60 (93.8%) | 0.48 (0.87) | 73 (100%) | 50 (68.5%) | 0.03 (0.16) |
| Hospital admission | Days | 64 (100%) | 57 (89.1%) | 16.7 (26.2) | 72 (98.6%) | 16 (21.9%) | 12.2 (10.2) |
| Hospital admission investigations | Investigations | 62 (96.9%) | 57 (89.1%) | 2.95 (2.58) | 70 (95.9%) | 16 (21.9%) | 0.31 (0.75) |
| Hospital admission medicines | Medicines | 61 (95.3%) | 57 (89.1%) | 3.82 (2.98) | 70 (95.9%) | 16 (21.9%) | 0.27 (0.92) |
| Surgery | Operations | 64 (100%) | 64 (100%) | 1.02 (0.12) | 73 (100%) | 0 (0%) | 0 (0) |
| Surgical implant | Implants | 59 (92.2%) | 59 (92.2%) | 1 (0) | 73 (100%) | 0 (0%) | 0 (0) |
| Surgical consumables | Consumables | 44 (68.8%) | 44 (68.8%) | 36.6 (13.4) | 73 (100%) | 0 (0%) | 0 (0) |
| Surgical antibiotics | Antibiotics | 49 (76.6%) | 43 (67.2%) | 0.9 (0.3) | 73 (100%) | 0 (0%) | 0 (0) |
| Discharge medications | Medicines | 57 (89.1%) | 57 (89.1%) | 2.56 (1.61) | 70 (95.9%) | 16 (21.9%) | 0.2 (0.71) |
| Discharge walking aids | Items | 64 (100%) | 57 (89.1%) | 0.3 (0.6) | 72 (98.6%) | 16 (21.9%) | 0 (0.1) |
| ***Follow-up after initial presentation*** | | | | | | | |
| Readmission | Days | 60 (93.8%) | 6 (9.4%) | 0.1 (0.3) | 71 (97.3%) | 1 (1.4%) | 0.01 (0.12) |
| Facility visits | Visits | 60 (93.8%) | 13 (20.3%) | 0.23 (0.46) | 71 (97.3%) | 7 (9.6%) | 0.1 (0.3) |
| Home visits | Visits | 51 (79.7%) | 3 (4.7%) | 0.08 (0.34) | 60 (82.2%) | 2 (2.7%) | 0.03 (0.18) |
| Medicines | Medicines | 60 (93.8%) | 12 (18.8%) | 0.23 (0.5) | 71 (97.3%) | 12 (16.4%) | 0.2 (0.47) |
| Home adaptation | Items | 60 (93.8%) | 0 (0%) | 0 (0) | 71 (97.3%) | 0 (0%) | 0 (0) |
| Equipment/walking aids | Items | 60 (93.8%) | 17 (26.6%) | 0.3 (0.5) | 70 (95.9%) | 14 (19.2%) | 0.23 (0.49) |
| ^ϕ^Refers to number of patients who received a service or used a resource. ^†^Home adaptation refers to any structural changes (e.g. installation of railings) to the patient’s home to enable them to manage better after hip injury. | | | | | | | |

**Figure S7.** Mean unadjusted costs aggregated over the main resource categories by care pathway and time point. In the sensitivity analysis, we recalculated unit costs for facility visits, admissions, and surgical procedures by applying an alternative cost allocation approach. Rather than using direct expenditures as in the base case analysis, we reallocated overhead and intermediate costs to final cost centres based on personnel numbers as the allocation basis.

Table S7. Mean unadjusted total costs aggregated over the main resource categories over the 12-months follow-up period. Costs are presented for the primary and sensitivity analysis. All costs are presented in 2024 United States dollars (US$).

| **Resource** | **Operated** | | **Not operated** | |
| --- | --- | --- | --- | --- |
| **Basecase analysis** | **n** | **Mean (95% CI)** | **n** | **Mean (95% CI)** |
| Prior care | 62 | 35 (6 - 78) | 73 | 50 (30 - 76) |
| Emergency department attendance | 63 | 83 (57 - 113) | 73 | 44 (26 - 69) |
| Outpatient department attendance | 63 | 5 (3 - 7) | 73 | 3 (1 - 4) |
| Hospital admission | 64 | 306 (175 - 474) | 73 | 34 (13 - 68) |
| Surgery | 64 | 494 (450 - 547) | 73 | 0 (0 - 0) |
| Surgical implant | 59 | 319 (287 - 355) | 73 | 0 (0 - 0) |
| Follow-up hospital admission | 60 | 6 (0 - 17) | 71 | 0 (0 - 0) |
| Facility visit | 59 | 23 (3 - 49) | 70 | 2 (0 - 5) |
| Home visit | 51 | 3 (0 - 6) | 60 | 0 (0 - 0) |
| Home adaptation | 60 | 0 (0 - 0) | 71 | 0 (0 - 0) |
| Equipment/walking aids | 60 | 4 (2 - 7) | 70 | 5 (2 - 8) |
| Medicines | 53 | 3 (0 - 9) | 67 | 2 (0 - 3) |
| Total costs | 37 | 1,086 (977 - 1,201) | 54 | 143 (96 - 200) |
| **Sensitivity analysis using alternative unit costs*** | | | | |
| Prior care | 62 | 35 (6 - 78) | 73 | 50 (31 - 74) |
| Emergency department attendance | 63 | 92 (66 - 120) | 73 | 50 (29 - 77) |
| Outpatient department attendance | 63 | 6 (3 - 8) | 73 | 3 (1 - 5) |
| Hospital admission | 64 | 335 (192 - 501) | 73 | 39 (16 - 80) |
| Surgery | 64 | 295 (242 - 365) | 73 | 0 (0 - 0) |
| Surgical implant | 59 | 319 (286 - 356) | 73 | 0 (0 - 0) |
| Follow-up hospital admission | 60 | 6 (0 - 16) | 71 | 0 (0 - 0) |
| Facility visit | 59 | 23 (4 - 49) | 70 | 2 (0 - 5) |
| Home visit | 51 | 3 (0 - 6) | 60 | 0 (0 - 0) |
| Home adaptation^†^ | 60 | 0 (0 - 0) | 71 | 0 (0 - 0) |
| Equipment/walking aids | 60 | 4 (2 - 7) | 70 | 5 (2 - 8) |
| Medicines | 53 | 3 (0 - 8) | 67 | 2 (0 - 3) |
| Total costs | 37 | 920 (799 - 1,061) | 54 | 154 (104 - 220) |
| ^†^Home adaptation refers to any structural changes (e.g. installation of railings) to the patient’s home to enable them to manage better after hip injury. *Unit costs for facility visits, admissions, and surgery were recalculated by reallocating overhead and intermediate costs to final cost centres using alternative allocation bases (personnel numbers) rather than direct expenditures. | | | | |

## Health outcomes

**Figure S8.** Unadjusted mean utilities as well as the mean EQ-VAS scores per participant for each treatment group at baseline and each timepoint over the 12-months follow-up period stratified by place of initial recruitment (public facility, private facility, or traditional bonesetter [TBS]). EQ-5D=European Quality of Life (EuroQol) 5 Dimensions, EQ-VAS = EuroQol Visual Analogue Scale.

|  |  |
| --- | --- |

**Figure S9.** Kernel density plot comparing the utility values distribution derived using the Ghanaian^2^ five-level (5L) EuroQol five-dimension (EQ-5D-5L) and the Zimbabwean^3^ EQ-5D-5L crosswalk value set. QALY=quality-adjusted life years

# References

1	Shepard DS, Hodgkin D, Anthony YE. Analysis of hospital costs: a manual for managers. World Health Organization, 2000.

2	Addo R, Mulhern B, Norman R, Owusu R, Viney R, Nonvignon J. An EQ-5D-5L Value Set for Ghana Using an Adapted EuroQol Valuation Technology Protocol. *Value Health Reg Issues* 2025; **45**: 101045.

3	van Hout B, Janssen MF, Feng Y-S, *et al.* Interim scoring for the EQ-5D-5L: mapping the EQ-5D-5L to EQ-5D-3L value sets. *Value Health* 2012; **15**: 708–15.
